# Genomic Diversity, Virulence Gene, and Prophage Arrays of Bovine and Human Shiga Toxigenic and Enteropathogenic *Escherichia coli* Strains Isolated in Hungary

**DOI:** 10.3389/fmicb.2022.896296

**Published:** 2022-07-05

**Authors:** Domonkos Sváb, Linda Falgenhauer, Tünde Mag, Trinad Chakraborty, István Tóth

**Affiliations:** ^1^Veterinary Medical Research Institute, Budapest, Hungary; ^2^Institute of Hygiene and Environmental Medicine and German Center for Infection Research (DZIF), Partner Site Giessen-Marburg-Langen, Justus Liebig University Giessen, Giessen, Germany; ^3^National Public Health Center, Budapest, Hungary; ^4^Institute of Medical Microbiology, German Center for Infection Research (DZIF), Partner Site Giessen-Marburg-Langen, Justus Liebig University Giessen, Giessen, Germany

**Keywords:** EHEC, STEC, aEPEC, cattle, WGS, prophage, comparative genomics, virulence gene array

## Abstract

*Escherichia coli* belonging to the enterohemorrhagic (EHEC), Shiga toxin-producing (STEC) and atypical enteropathogenic (aEPEC) pathotypes are significant foodborne zoonotic pathogens posing serious health risks, with healthy cattle as their main reservoir. A representative sampling of Hungarian cattle farms during 2017–2018 yielded a prevalence of 6.5 and 5.8% for STEC and aEPEC out of 309 samples. The draft genomes of twelve STEC (of them 9 EHEC) and four aEPEC of bovine origin were determined. For comparative purposes, we also included 3 EHEC and 2 aEPEC strains of human origin, as well four commensal isolates and one extraintestinal pathogenic *E. coli* (ExPEC) obtained from animals in a final set of 26 strains for a WGS-based analysis. Apart from key virulence genes, these isolates harbored several additional virulence genes with arrays characteristic for the site of isolation. The most frequent insertion site of Shiga toxin *(stx)* encoding prophages was *yehV* for the Stx1 prophage and *wrbA* and *sbcB* for Stx2. For O157:H7 strains, the locus of enterocyte effacement pathogenicity island was present at the *selC* site, with integration at *pheV* for other serotypes, and *pheU* in the case of O26:H11 strains. Several LEE-negative STEC and aEPEC as well as commensal isolates carried additional prophages, with an average of ten prophage regions per isolate. Comparative phylogenomic analysis showed no clear separation between bovine and human lineages among the isolates characterized in the current study. Similarities in virulence gene arrays and close phylogenetic relations of bovine and human isolates underline the zoonotic potential of bovine aEPEC and STEC and emphasize the need for frequent monitoring of these pathogens in livestock.

## Introduction

Shiga toxin-producing *Escherichia coli* (STEC) are an intestinal pathotype of *E. coli,* currently comprising more than 400 serotypes (Blanco et al., 2004) their common feature being the production of Shiga toxin (also called Vero-cytotoxin, Stx), a potent cytotoxin inhibiting protein synthesis in the affected cell lines. A highly virulent subset of STEC strains termed enterohemorrhagic *E. coli* (EHEC) produce intimin apart from Stx, an adhesin that is responsible for attaching/effacing lesion on intestinal epithelial cells (Kaper et al., 2004). In addition to hemorrhagic colitis, EHEC cause a severe systemic complication called hemolytic-uremic syndrome (HUS), which results in acute renal failure in affected patients (Bielaszewska and Karch, 2005).

The primary reservoir of EHEC isolates is cattle, with isolates being present in the feces of the animals (Newell and Ragione, 2018). Human infection can result from direct contact with the animals or with their feces but occurs more frequently as a result of the consumption of undercooked meat, or of fresh vegetables, drinking water or meat contaminated with cattle feces (Chekabab et al., 2013; Terajima et al., 2017). The domestic animal reservoir, the severity of the disease, and low infective dose (<100 cells/person, Pulz et al., 2003), make EHEC serious and deadly zoonotic and foodborne pathogens. Enteropathogenic *E. coli* (EPEC) is the first described pathotype of *E. coli* and closely related to EHEC, responsible for a high proportion of children’s diarrhea mainly in countries with poor infrastructures (Ochoa and Contreras, 2011). Its key virulence factor is intimin, but unlike EHEC they do not produce Stx (Croxen et al., 2013). A subset of EPEC termed atypical EPEC (aEPEC) which are isolated from both humans and animals, do not carry the gene encoding the bundle-forming pilus *(bfp)*. These isolates are genetically more closely related to EHEC, and it was recently suggested that subsets of aEPEC strains are possible progenitors of EHEC strains (Ferdous et al., 2015; Eichhorn et al., 2018).

An important feature of STEC, EHEC and EPEC is that their major virulence factors are carried on mobile genetic elements (MGE). Genes encoding *stx* are generally carried by lambdoid prophages, which are frequently inducible and transmissible (Ohnishi et al., 2002; Asadulghani et al., 2009; Rahman et al., 2018). In the case of both EHEC and EPEC, the *eae* gene encoding intimin is part of a 35–43 kb long pathogenicity island (PAI) termed locus of enterocyte effacement (LEE; reviewed by Jores et al., 2004), which carries genes encoding the type III secretion system, as well as effector proteins including translocated intimin receptor (*tir*) gene.

Highly virulent EHEC strains comprise members of the O157:H7 serotype (Hayashi, 2001; Perna et al., 2001; Paletta et al., 2020). Monitoring cattle for the presence of these strains has become a necessity, in order to understand the factors and risks associated with the transmission of EHEC O157 strains from animals to humans, as well as for epidemiological tracing.

The availability of affordable whole-genome sequencing (WGS) enables rapid assessment of genetic information encoded by a large set of strains, shedding light on of yet unknown genetic variants, additional virulence genes, and associated MGEs (Kulasekara et al., 2009). Genomic studies characterizing *E. coli* O157 strains revealed a remarkable genetic diversity of the serogroup (Ogura et al., 2006; Eppinger et al., 2011) especially regarding their prophage content (Shaaban et al., 2016; Sharma et al., 2019). A WGS-based study revealed that a specific subset of strains belonging to the serogroup, are frequently carried by cattle and responsible for the serious human outbreaks (Lupolova et al., 2016). It is therefore necessary to investigate cattle herds for the presence of these highly virulent strains and use the genome-based information to explore their genetic variability and assess their zoonotic potential. As the example of the recent outbreak caused by O80:H2 strains representing hybrid pathotype of STEC and ExPEC has shown (Nüesch-Inderbinen et al., 2018), the emergence of new, highly virulent pathotypes or clones is an ever-present possibility.

A previous explorative sampling of Hungarian cattle farms, which provided the first information on the prevalence of pathogenic strains of the O157 serogroup among cattle herds in Hungary used comprehensive PCR-based virulence profiling, and a classification based on pulsed-field gel electrophoresis (PFGE) together with multi-locus sequence typing (MLST) profiling of the isolated strains (Tóth et al., 2009). In addition to EHEC O157:H7, EPEC isolates of the same serotype, as well as those with other H types and atypical virulence gene repertoires are also present in cattle (Sváb et al., 2016).

The main aim in this study was to obtain further information regarding the prevalence of potentially zoonotic *E. coli* on Hungarian cattle farms, with a focus on the O157 serogroup and the STEC, EHEC and EPEC pathotypes. Using a WGS-based approach, we aimed to reveal potentially new genetic variants, virulence gene sets and MGEs, as well as to compare these data obtained from cattle to those of strains isolated in the earlier study together with those originating from human patients. In addition to assessing the occurrence of strains with high zoonotic potential, we also aimed to reveal potentially new genotypes, comprehensively map their MGEs, as well as their integration sites in the respective genomes.

## Materials and Methods

### Sample Collection and Bacterial Strains

Samples were collected from cattle farms throughout Hungary, both from the feces and milk of animals, as well as samples representing the environment, collected from the barn floor at selected farms. A total of 309 samples, comprising of fecal samples (*n* = 215), and milk samples (*n* = 81) from 215 healthy cattle, along with 13 samples from the farm environment were taken. Sampling was performed on 18 cattle farms representing different regions in Hungary involved in both dairy and meat production between 2017 and 2018. Samples were either processed immediately or stored at −70°C until required.

Strains were isolated using the ISO protocol 16,654:2001 recommended for the isolation of *E. coli* serotype O157 strains (Standard British and BSEN ISO, 2001). Briefly, following pre-enrichment in tryptone-soy broth (TSB) supplemented with bile salts, samples underwent immunomagnetic separation (IMS) with DynaBeads anti-*E. coli* O157 kit (Applied Biosystems) according to the manufacturer’s instruction. Samples were spread on sorbitol MacConkey (SMAC) agar plates, with cefixime as an additive selective agent.

From each sample, four sorbitol non fermenting (SNF) and one sorbitol-fermenting (SF) colony were picked from the SMAC agar plates and cultured in non-selective Luria–Bertani (LB) broth in 96-well plates. Every colony, which was positive for either of the key virulence genes, was stored and handled as a separate isolate in LB broth supplemented with 30% glycerol at −70°C.

Additionally, for comparative genomic purposes, we also included five STEC and EPEC strains isolated from human patients in 2018. One bovine EHEC and three bovine EPEC strains isolated by Tóth et al. (2009) were included in the sequencing as well.

Strains selected for further studies were grown overnight in LB broth, then the culture was mixed with 30% volume of sterile glycerol and stored at −70°C.

### Detection of Key Virulence Genes

To detect the presence of STEC and EPEC among isolates, PCR screening of *stx* (Scheutz et al., 2012) and *eae* (China et al., 1996) genes was performed for all isolates, from which those for sequencing based were selected based on detected virulence profile.

### Serotyping of Selected Isolates

Preliminary detection of the O157 antigen was performed with Oxoid latex agglutination test according to the manufacturer’s instruction. In all other cases of strains selected for genome sequencing serotyping was performed with O-specific and H-specific antisera following standard protocols (Ørskov and Ørskov, 1984). After obtaining whole-genome sequences, *in silico* serotyping was performed using SerotypeFinder 2.0 (Joensen et al., 2015).

### Genome Sequencing

Seventeen bovine strains isolated in the current study were chosen for whole-genome sequencing (WGS). For comparative purposes, we also sequenced one EHEC O157:H7 as well as three EPEC strains isolated in the earlier study of Tóth et al. (2009), as well as two EPEC and three STEC strains of human origin, thus altogether 26 WGS were obtained. Isolates were chosen for WGS to represent not only different samples and sampling sites, but also distinctive arrays of key virulence genes. Strains containing either only one *stx* gene, or both *stx1* and *stx2* were selected as well. Genomic DNA of the strains was isolated with the ZymoResearch Quick-DNA Fungal/Bacterial Miniprep Kit according to the manufacturer’s instructions. Sequencing was performed on an Illumina NextSeq system as follows. Sequencing libraries were generated using Illumina Nextera XT Kit (Cat.Num.: FC-131-1096, Illumina, Eindhoven, Netherlands) as per manufacturer’s instructions. DNA sequencing was performed on an Illumina NextSeq 500 machine (Illumina, Eindhoven, Netherlands) with 2x150nt read length chemistry (mid-output/high output flow cells). Sequencing quality assessment and assembly was performed using ASA^3^P (Schwengers et al., 2020), including the SPAdes Software for assembly (Bankevich et al., 2012). The average read length was 122 nt and the coverage ranged from 62.14x to 171. 88x.The genomes were assembled into contigs ranging from 50 to 1947 in number (Table 1). All sequence data are available in GenBank through Bioproject no. PRJNA764596, in BioSample IDs SAMN21509413-SAMN21509438 and Sequence Read Archive nos. SRR15970245-SRR15970246.

**Table 1:** Summary of sequencing results for strains with their whole-genome sequenced in this study.

Strain	Source	Phylogenetic group	Pathotype	Serotype	Sequence type	Stx	*stx1* subtype	*stx2* subtype	*eae* subtype	*LEE*	*pO157*	*ehx*	Coverage	Number of contigs
52	bovine	E	EHEC	O157:H7	11	1,2	a	a	γ1	yes	yes	yes	105.74	304
65	bovine	E	aEPEC	O157:H7	11		N/A	N/A	γ1	yes	yes	yes	79.75	255
34-2	bovine	A	aEPEC	O90:H40	10		N/A	N/A	θ	yes	/	/	151.25	185
64-2	bovine	E	aEPEC	O90:H40	10		N/A	N/A	θ	yes	yes	yes	89.98	766
1511	human	A	aEPEC	O-:H26	189		N/A	N/A	γ1	yes	/	/	91.40	1947
1512	human	E	EHEC	O157:H7	11	1,2	a	a	γ1	yes	yes	yes	76.82	358
1513	human	E	EHEC	O157:H7	11	2	N/A	c	γ1	yes	yes	yes	62.14	387
1514	human	E	EHEC	O157:H7	11	1,2	a	c	γ1	yes	yes	yes	97.30	385
1515	human	A	aEPEC	O157:H16	10		N/A	N/A	ε1	yes	/	/	73.22	184
KP1A	bovine	B1	EHEC	O26:H11	21	1	a	N/A	β1	yes	/	yes	118.43	526
KP2E	bovine	B1	EHEC	O26:H11	21	1	a	N/A	β1	yes	/	yes	120.51	549
KP4E	bovine	B1	EHEC	O26:H11	21	1	a	N/A	β1	yes	/	yes	114.44	525
Emőd10B	bovine	U/cryptic	commensal	O79:H2	4,419		N/A	N/A	N/A	/	/	/	125.17	50
MOS2/3A	bovine	B1	aEPEC	O182:H25	300		N/A	N/A	ζ3	yes	/	yes	171.88	309
MGKF1	bovine	E	EHEC	O157:H7	11	1,2	a	a	γ1	yes	yes	yes	97.12	354
ML2/4E	bovine	B1	commensal	not typeable	345		N/A	N/A	N/A	/	/	/	122.03	94
ML2/7C	bovine	A	STEC	O-:H4	10	2	N/A	d	N/A	/	/	/	105.94	253
ML2/9C	bovine	D	ExPEC	O102:H6	405		N/A	N/A	N/A	/	/	yes$	100.30	375
ML5/10A	bovine	B1	STEC	O-:H7	278	1,2	a^*^	c	N/A	/	/	/	119.13	459
ML5/5A	bovine	B1	STEC	O8:H21	1794	1,2	a	c	N/A	/	/	/	94.40	659
ML5/6D	bovine	B1	commensal	O2/O50:H8	6,445		N/A	N/A	N/A	/	/	/	120.26	194
ML6/11A	bovine	E	EHEC	O157:H7	11	1,2	a	c	γ1	yes	yes	yes	132.10	338
ML6/1A	bovine	E	EHEC	O157:H7	11	1,2	a	c	γ1	yes	yes	yes	110.08	353
ML6/20A	bovine	E	EHEC	O157:H7	11	1,2	a	c	γ1	yes	yes	yes	101.50	338
ML6/20E	bovine	B1	commensal	O1:H7	101		N/A	N/A	N/A	/	/	/	117.05	151
ML6/4B	bovine	E	EHEC	O157:H7	11	1,2	a	c	γ1	yes	yes	yes	111.72	341

*Strains described in Tóth et al. (2009) are marked with yellow, strains of human origin described in the current study are marked with pink, and strains isolated from bovine sources in the current study are marked with light blue.*

* *only subunit A is present, not B.*

*$ehxB is missing.*

### Virulence, Antibiotic Resistance Gene and Prophage Searches

Virulence gene search was performed using VirulenceFinder (Joensen et al., 2015) and Virulence Factor Database (VFDB, Liu et al., 2022) antimicrobial resistance genes were searched for using ResFinder (Zankari et al., 2012), and prophage regions were searched by PHASTER (Arndt et al., 2016). Subtypes of intimin genes (*eae*) were determined using tblastN and the references depicted by Yang et al. (2020). The presence of the pO157 plasmid (accession number AB011549.2) was determined using blastN.

The intact or disrupted state of the characteristic *stx* prophage and LEE integration sites listed by Bonanno et al. (2015) and Saile et al. (2018), respectively, was checked by blastN of the respective empty integration sites. The flanking regions of occupied integration sites were analyzed using PHASTER to determine the nature of the respective phage or LEE island (e.g., whether this was a putative *stx*-encoding prophage or not).

### Phylogenetic Grouping and MLST Analysis

Determination of the phylogenetic group was performed *in silico* using the online software tool “*In Silico* Clermont Phylotyper”^1^ based on the protocol of Clermont et al. (2013) that targets several housekeeping genes and additional regions. MLST was performed according to the Wirth scheme utilizing the nucleotide sequence of seven housekeeping genes (Wirth et al., 2006). A core-genome-based maximum likelihood phylogenetic tree was constructed with ParSNP implemented in the HarvestSuite program package (Treangen et al., 2014) using the genome set published by Arimizu et al. (2019). The full dataset is listed in Supplementary Table 1.

## Results

### Prevalence of Pathotypes Among the Isolates

To reveal the incidence of STEC and EPEC in Hungarian cattle herds, we sampled 215 animals from 18 cattle farms throughout Hungary, taking fecal samples from all animals, and milk samples from a total of 81 animals, as well as 13 environmental samples. A collection of 184 *E. coli* strains isolated from bovine sources during 2013–2017 was also examined.

The *stx* genes were detected in 20 out of 309 samples (6.5%) and from the *stx*-positive samples a total of 47 STEC isolates were identified, as multiple colonies were stored from each sample. Out of these, 18 also carried the *eae* gene, and they were classified as EHEC; these EHEC isolates represented nine samples altogether. A further 18 samples (5.8%) contained isolates carrying *eae* without *stx,* and altogether 44 such genotype isolates, classified as EPEC, were identified.

Draft genomes from a total of 12 bovine STEC, (out of them 9 EHEC), 4 bovine EPEC, 3 human EHEC, and 2 human EPEC strains, as well as 5 *stx-, eae- E. coli* strains labeled as “commensal” were analyzed. The bovine strains represented strains isolated in the current study as well as those from the earlier collection by Tóth et al. (2009). Sequencing data revealed that in addition to the O157:H7 strains 34 and 64 characterized in that study, the original samples contained further *E. coli* strains, named 34-2 and 64-2, respectively. In the present study, these novel isolates were characterized. Two human and one bovine isolate was negative for *stx* genes, that had previously been detected by PCR, but as they were *eae-*positive, they were reclassified as EPEC. In light of its sequence type and virulence gene array detailed below, one of the commensal strains was reclassified as ExPEC (Table 1).

The sizes of the genomes ranged from 4,595,164 to 5,962,783 bp, their GC content ranged from 50 to 51%.

### Sequence Types, Phylogenetic Relations and Serotypes

To reveal the phylogenetic relationship of the isolates the main phylogenetic groups and the sequence types (STs) were determined (Table 1). Twelve different STs were detected. The most common ST was ST11 (*n* = 10), followed by ST10 (*n* = 4) and ST21 (*n* = 3). The remaining nine STs (Table 1) were represented by single isolates.

**Figure 1:**
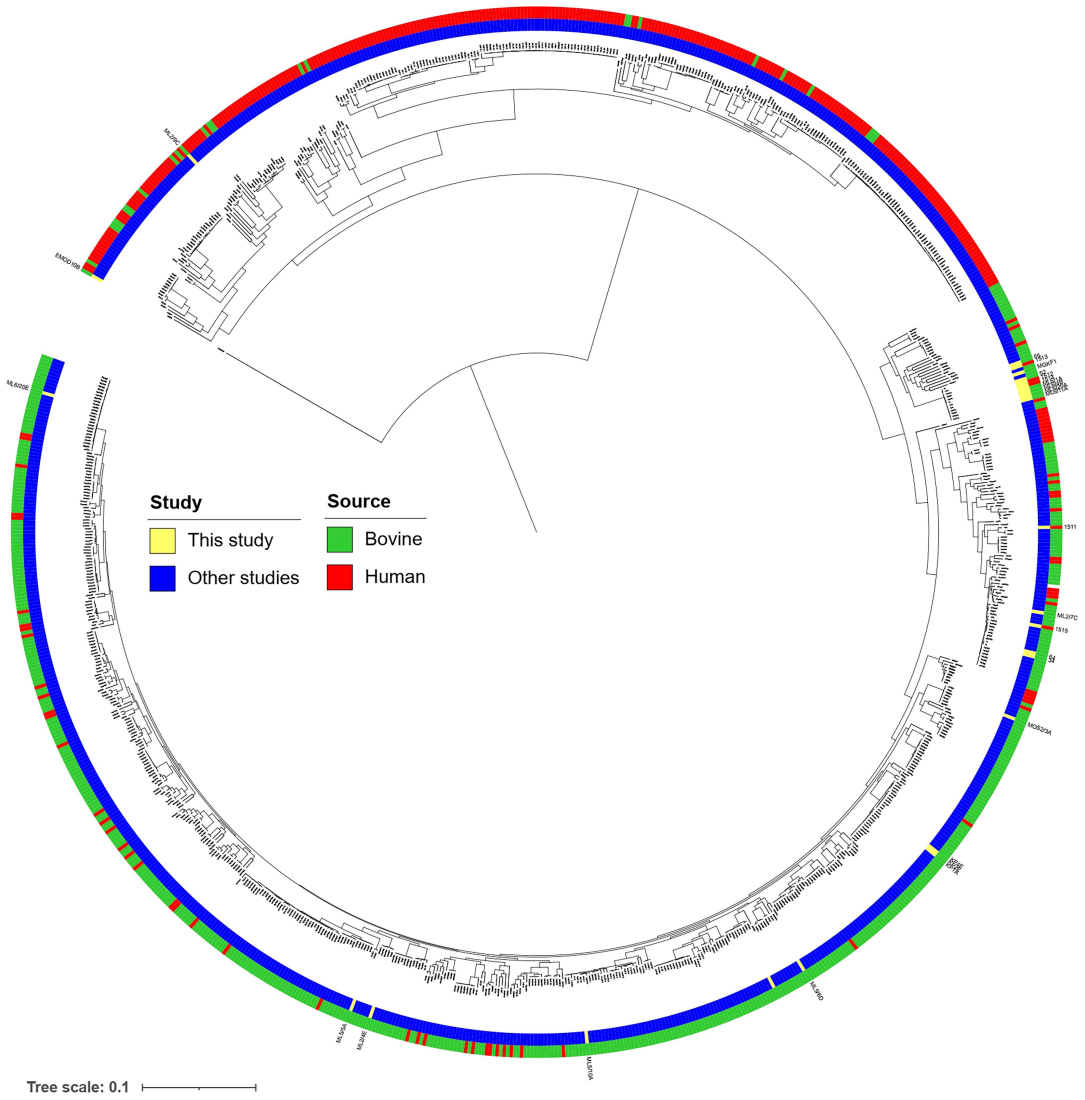
Core gene-based neighbor-joining tree of the 937 bovine and human commensal isolates with 197 human clinical isolates described in Arimizu et al. (2019), complemented with strains with WGS determined in the current study, marked with yellow.

The core-genome-based phylogenetic relations were compared to a previously compiled global set of EPEC and STEC strains and is visualized in Figure 1.

All strains were serotyped using *in silico* tools (Table 1). The O157:H7 serotype was represented by EHEC strain 52 and EPEC strain 65 of earlier isolation (Tóth et al., 2009), as well by three strains of human origin (1,512, 1,513, 1,514), and by strains with the “ML6” designation, which were isolated from a single farm, but from different animals (Table 1), except for the commensal strain ML6/20E (Table 1). The STs of the O157 serogroup strains were remarkably uniform independently of their serotypes, as all were of ST11.

The “KP” strains (originating from one farm) were of the O26:H11 serotype and belonged all to ST21.

None of the remaining isolates represented any of the “big six” serogroups. This was true for ST10 isolates as well, it was represented by three different serotypes (O90:H40, O157:H4/H16). ST10 was represented by two bovine strains of earlier isolation (34-2, 64-2), one newly isolated bovine strain (ML2/7C) and one human (1515) strain. The serogroup of STEC strains ML2/7C and ML5/10A was O-:H4 and O-:H7, respectively. The human strain 1,511 depicted the O-:H26 serogroup. The commensal isolates exhibited various serotypes, the ExPEC ML2/9C represented serotype O102:H6.

### Key Virulence Genes and Their Genetic Background

Characteristic Stx prophage, lambdoid phages and LEE integration sites in various strains were observed (Table 2).

**Table 2:** Integration sites used by the Stx prophages and locus of enterocyte effacement (LEE) pathogenicity island in the strains with their whole-genome sequences determined in this study.

Strain	Pathotype	Serotype	*stx* genes carried	Stx1 prophage insertion site	Stx2 prophage insertion site	LEE island insertion site
52	EHEC	O157:H7	1,2	*yehV*	*wrbA*	*selC*
65	aEPEC	O157:H7	–	N/A	N/A	*selC*
1511	aEPEC	O-:H26	–	N/A	N/A	*pheV*
1512	EHEC	O157:H7	1,2	*yehV*	*wrbA*	*selC*
1513	EHEC	O157:H7	2	N/A	*sbcB*	*selC*
1514	EHEC	O157:H7	1,2	*yehV*	*sbcB*	*selC*
1515	aEPEC	O157:H16	–	N/A	N/A	*pheV*
34-2	aEPEC	O90:H40	–	N/A	N/A	*pheV*
64-2	aEPEC	O90:H40	–	N/A	N/A	*pheV*
Emőd10B	commensal	O79:H2	–	N/A	N/A	N/A
KP1A	EHEC	O26:H11	1	*wrbA*	N/A	*pheU*
KP2E	EHEC	O26:H11	1	*wrbA*	N/A	*pheU*
KP4E	EHEC	O26:H11	1	*wrbA*	N/A	*pheU*
MGKF1	EHEC	O157:H7	1,2	*yehV*	*wrbA*	*selC*
ML2/4E	commensal	not typeable	–	N/A	N/A	N/A
ML2/7C	STEC	O-:H4	2	N/A	not determined	N/A
ML2/9C	ExPEC	O102:H6	–	N/A	N/A	N/A
ML5/10A	STEC	O-:H7	1,2	*yehV*	*wrbA*	N/A
ML5/5A	STEC	O8:H21	1,2	*yecE*	*wrbA*	N/A
ML5/6D	commensal	O2/O50:H8	–	N/A	N/A	N/A
ML6/11A	EHEC	O157:H7	1,2	*yehV*	*sbcB*	*selC*
ML6/1A	EHEC	O157:H7	1,2	*yehV*	*sbcB*	*selC*
ML6/20A	EHEC	O157:H7	1,2	*yehV*	*sbcB*	*selC*
ML6/20E	commensal	O1:H7	–	N/A	N/A	N/A
ML6/4B	EHEC	O157:H7	1,2	*yehV*	*sbcB*	*selC*
MOS2/3A	aEPEC	O182:H25	–	N/A	N/A	*pheV*

Thirteen isolates harbored a *stx1*, and twelve isolates carried the *stx2* gene. Ten isolates including two human isolates carried both *stx* genes; eight were of the O157:H7 serotype and the MLST type ST11. Three different Stx1-prophage (*yecE*, *yehV*, *wrbA*) and two different Stx2-prophage integration sites were detected (*sbcB*, *wrbA*, Table 2).

**Figure 2:**
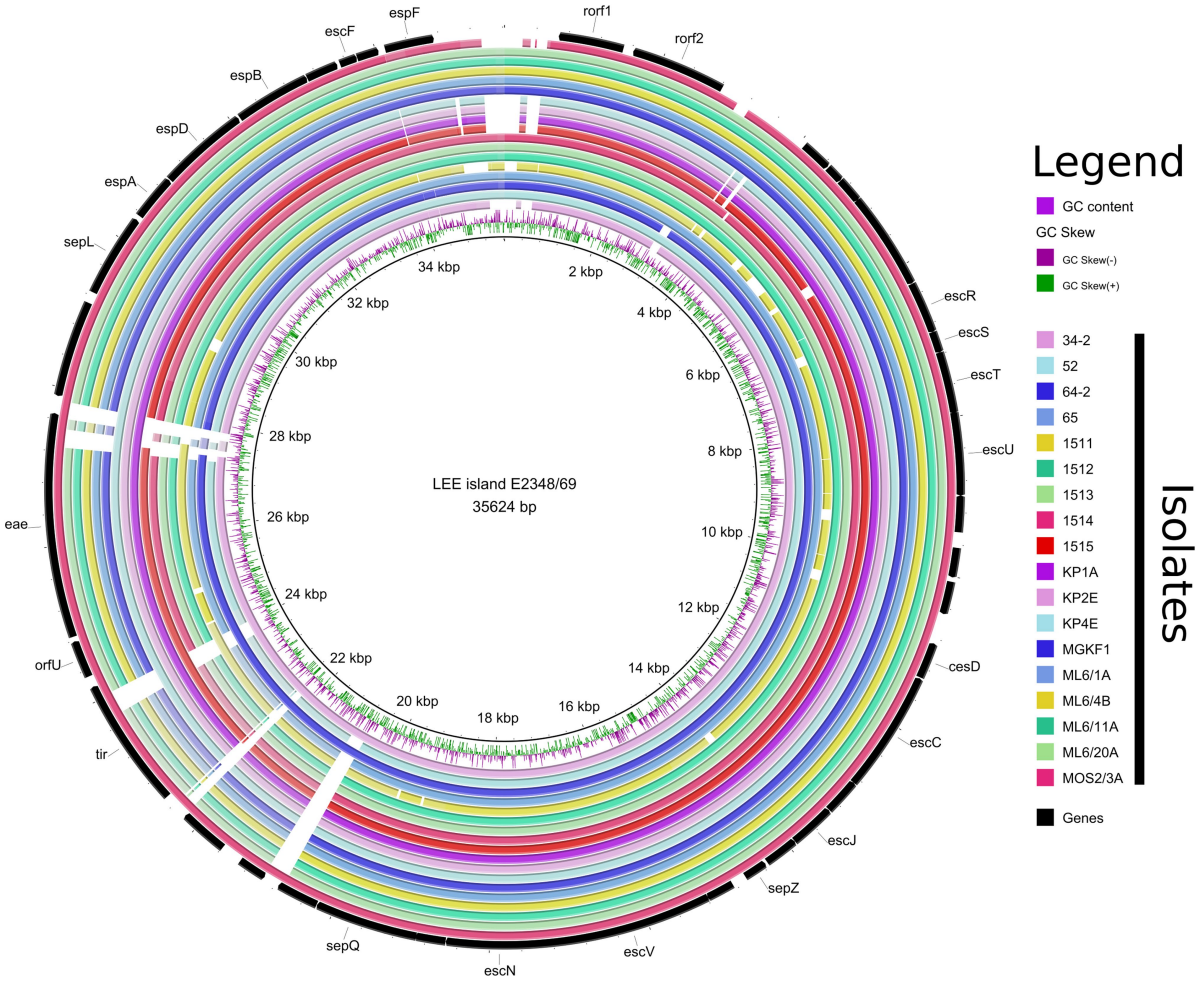
Representation of the LEE pathogenicity islands of all EPEC and EHEC strains with WGS determined in this study. The regions are shown circularized for a more compact view.

A LEE island was detected in 18/26 whole-genome sequenced isolates. It was integrated at three different sites (*selC*, *pheV*, *pheU*, Table 2). The summary of the integration sites of Stx prophages and LEE islands in the investigated strains is shown in Table 2. In the case of human strains, the Stx1 prophage and the LEE tended to be integrated into the same, frequently used sites (*yehV* and *selC,* respectively), while the bovine strains were more diverse in this regard (*yehV*, *wrbA* and *yecE* for the Stx1 prophage, *selC*, *pheU* and *pheV* for LEE sites), with the tendency that strains from the same farm and with the same serotype showed similar integration patterns for these two MGEs. The detailed structure of the LEE of all WGS strains is shown in Figure 2 in a circularized form. The LEE island differed in the strains in several intergenic regions and in the two genes *eae* and *tir,* in which for some isolates no homology could be detected at the end of the gene (Figure 2).

### Additional Virulence Genes

Using VirulenceFinder and VFDB, we identified a total of 41 additional virulence-related genes in the genomes apart from the *stx1* and *stx2* genes (Table 3; Supplementary Table 2) as well as of the LEE-associated effectors. All *eae*-positive strains carried the *tir* gene, but only six strains harbored the *tccP.* Subtyping of the intimin gene (*eae*) revealed the presence of five different intimin subtypes (β1, γ1, ε1, ζ3, θ).

**Table 3:** Summary of additional virulence factors encoded by strains with WGS determined in this study.

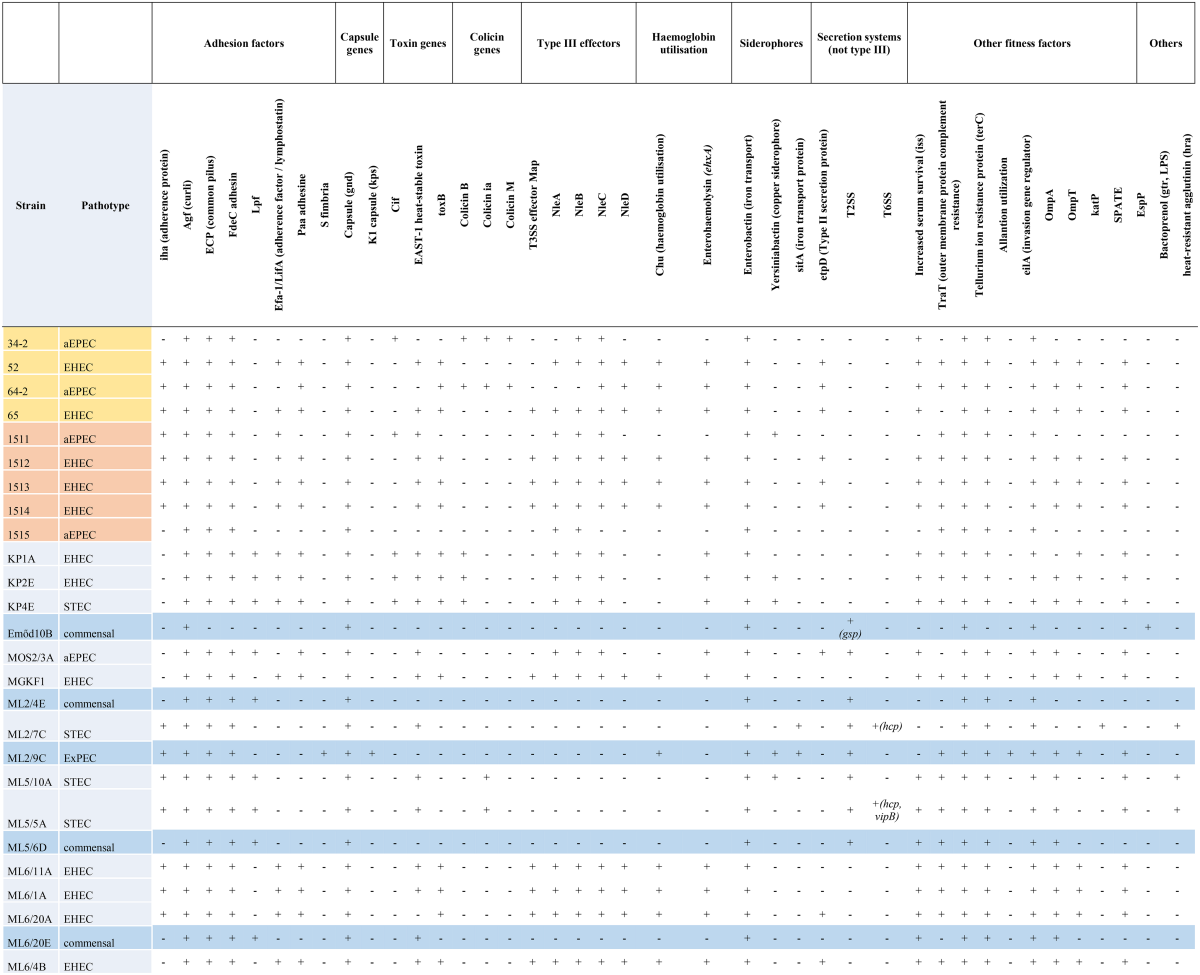

*Strains described in Tóth et al. (2009) are marked with yellow, strains of human origin described in the current study are marked with pink, and strains isolated from bovine sources in the current study are marked with light blue. The commensal and ExPEC strains are highlighted by darker blue.*

All strains of the serotype O157:H7 also carried the pO157 plasmid that included several classical pO157 plasmid effectors, e.g., the enterohemolysin-encoding operon (*ehxABCD*). The bovine ExPEC strain ML2/9C carried pO157 that also included *katP*.

Other widely occurring virulence-related genes of note included the *astA* encoding the enteroaggregative *E. coli* heat-stable enterotoxin 1 (EAST1), the *iha* and *lpfA* encoding adhesins, the *iss* gene that encodes a serum survival factor as well as the gene encoding the iron siderophore enterobactin.

Based on the virulence gene content, four different pathotypes could be assigned to the whole-genome sequenced isolates (EHEC, EPEC, STEC and ExPEC). No strain harbored the genes encoding bundle-forming pilus (*bfp*), therefore all the EPEC strains with their WGS determined represented the atypical EPEC (aEPEC) strains. The most common pathotype was EHEC (*n* = 12), followed by aEPEC (*n* = 6) STEC (*n* = 3) and ExPEC (*n* = 1). The remaining four isolates were classified as commensal *E. coli*. A summary of additive virulence genes is shown in Table 3, while the detailed virulence-related gene content identified in each strain is given in Supplementary Table 2. The detailed comparative map of LEE islands highlighting the effector genes is shown in Figure 2.

### Antimicrobial Resistance Genes

Five of 26 isolates sequenced carried antibiotic resistance genes (Table 4). The highest number of antibiotic resistance genes were found in an isolate lacking *stx* and *eae*, ML2/9C. It was classified as multidrug-resistant (MDR), because it harbored a total of 10 genes encoding resistance toward eight different groups of antimicrobials, including fluoroquinolones (*aac(6’)Ib-cr*), aminoglycosides (*aac(3)-IIa, aadA5*), macrolides (*mph(A)*), sulfonamides (*sul1*), tetracyclines (*tet(B)*), phenicols (*catB3*), and beta-lactams (*bla*_CTX-M-15_, *bla*_OXA-1_, Table 4). The *bla*_CTX-M-15_ gene represents an extended-spectrum beta-lactamase (ESBL). The other four isolates carrying antibiotic resistance genes (34-2, 64-2, 1,515, ML2/7C) harbored between one and three antibiotic resistance genes encoding resistance to aminoglycosides (*strA/strB*), sulfonamides (*sul2*) and/or beta-lactams (*bla*_TEM-1B_).

**Table 4:** Summary of identified antibiotic resistance genes in strains with their whole-genome sequences determined in this study.

Strain	Pathotype	Aminoglycoside	Beta-lactam	Macrolide	Sulfonamide	Trimethoprim	Tetracycline	Phenicol	Fluoroquinolone and aminoglycoside
34-2	aEPEC	strA,strB	–	–	sul2	–	–	–	–
64-2	aEPEC	strA,strB	–	–	sul2	–	–	–	–
1515	aEPEC	strA,strB	bla_TEM-1B_	–	–	–	–	–	–
ML2/7C	STEC	–	–	–	sul2	–	–	–	–
ML2/9C	ExPEC	*aac(3)-IIa,aadA5*	*bla_CTX-M-15_, bla_OXA-1_*	*mph(A)*	*sul1*	*dfrA17*	*tet(B)*	*catB3*	*aac(6’)Ib-cr*

*Strains described in Tóth et al. (2009) are marked with yellow, strains of human origin described in the current study are marked with pink, and strains isolated from bovine sources in the current study are marked with light blue.*

### Prophage Pool

Following a PHASTER search, in each genome of the investigated strains we found up to 5 intact prophages, between 2 and 12 incomplete prophage sequences, as well as up to 2 probable prophage-like sequences (Supplementary Table 3). We could not identify intact prophages in strains 65 and 1,513. A limitation of the results was that gapped genomes were used for the analysis; therefore, it is possible that the real number of prophages was higher in each strain than what was detected. The most prophage-rich genome was that of strain MOS2/3A with 4 complete, 10 incomplete and 2 phage-like regions. The length of the complete prophage regions ranged from 6.1 to 222 kb, and the percentage of complete prophages of the strains ranged from 43.80 to 56.15%. A summary of all detected prophages with accession numbers of their closest homologue sequences available in GenBank are listed in Supplementary Table 3.

## Discussion

In this study, representative sampling of Hungarian cattle farms was carried out to detect STEC, EHEC and EPEC. The prevalence of aEPEC isolates was 8.3% (19/228), while that of STEC it was 8.7% (20/228), and that of EHEC alone 3.9% (9/228). These values are congruent with earlier findings and suggest that Hungary has an occurrence of STEC, EHEC and EPEC isolates that is comparable to other countries of the developed world (Hussein, 2007; Tóth et al., 2009; Dong et al., 2017). It must be noted however, that our method for isolation was optimized for the isolation of strains of the O157 serogroup, therefore strains of all pathotypes representing other serogroups could be underrepresented in our collection. In the earlier study of Tóth et al. (2009), twice as many EPEC isolates were detected as compared to EHEC among the O157:H7 serotype. Two such aEPEC isolates (64-2 and 65) from the earlier study had highly similar virulence gene arrays to EHEC isolates in the current study as well as from humans suggesting that there are several aEPEC ancestral isolates of EHEC, essentially only lacking the Stx prophage (Ferdous et al., 2015; Eichhorn et al., 2018). At the same time, it cannot be excluded that these aEPEC strains might have lost their Stx prophage(s) (Bielaszewska et al., 2008). As none of the EPEC in our current study carried the *bfp* gene, all of them fall into the category of aEPEC. It is also apparent that such strains are present in similar prevalence among Hungarian cattle like the STEC strains. There was a tendency of strains originating from the same farms to harbor key virulence gene carrying MGEs in the same integration sites, as well as similar sets of additive virulence genes and prophages.

The genomic variability of the investigated pathotypes is most significant with respect to virulence gene repertoires. Regarding the *stx* genes, only the *stx1a* subtype was found in all *stx1+* strains, while *stx2*, where present, showed greater variability (*stx2a*, *n* = 3; *stx2c*, *n* = 8; *stc2d*, *n* = 1; Table 1). Almost all *stx2* subtype *a* and *c* were detected in O157:H7 strains (2/3 and 6/8, respectively), whereas the single subtype *d* was detected in an O157:H4 serotype isolate (ML2/7C, ST1794). In the case of the strain ML2/7C the integration site for the Stx2 prophage could not be identified, as all the other characteristic integration sites were either intact or occupied by other prophages. This finding suggests the presence of an alternative, hitherto unknown integration site for the Stx2 prophage in this strain, a possibility that has been recently raised for STEC strains (Henderson et al., 2021). It is also noteworthy that the aEPEC strains had at least two of the typical Stx prophage integration sites unoccupied, therefore they can be considered as possible recipients of this MGE and have potential of becoming EHEC (Tables 3, 4).

The *eae* genes represented five types (β1, γ1, ε1, ζ3, θ). The most common subtype was γ1 (*n* = 11) and was detected almost exclusively in O157:H7 strains (exception: 1511,: H26) irrespective of their source (human/animal). The second most common subtype was β1, which was detected only in O26:H11 strains with the “KP” designation, originating from the same farm. These findings confirm the notion that the carriage of intimin subtypes is associated to serotypes of EHEC and EPEC (Oswald et al., 2000; Tarr and Whittam, 2002).

A notable difference could be observed in the integration sites used by the LEE when comparing aEPEC and EHEC strains. In EPEC strains, LEE was almost exclusively (5/6) integrated in *pheV* (exception: *selC*), while for EHEC strains, LEE was integrated either in *selC* (*n* = 9) or *pheU* (*n* = 3). This difference could be attributed to the different clonal lineages of strains representing the two pathotypes (Ingle et al., 2016).

Besides the *stx* genes and LEE-encoded effectors, additional virulence-related genes were identified all the strains. Except for ML2/7C, ML5/5A and ML5/10A, all EHEC strains as well as the aEPEC strain 65 carried the *ehxA* encoding enterohemolysin, which suggests to the presence of the pO157 plasmid in their case. This distribution is also congruent with the results of Arimizu et al. (2019), who found that STEC strains retained *ehxA* more frequently than *stx*-negative ones. The presence of at least one pO157-associated effector gene suggests the carriage of this virulence plasmid (Lim et al., 2010) by all the WGS strains in this study, which belonged to the O157:H7 serogroup. The majority of the aEPEC and EHEC strains harbored effectors of the OI-122 genomic island (*efa1* and the non-LEE effectors *nleABCD*) widespread in these pathotypes (Karmali et al., 2003; Konczy et al., 2008) as well as additional adhesines, siderophores and other fitness factors (Table 3). Out of these, the LEE-negative strains (both STEC, commensal and the ExPEC ML2/9C) tended to carry alternative adhesin encoding genes: the *iha,* genes (Tarr and Whittam, 2002), and *lpfA*, the latter being widespread in various intestinal *E. coli* pathotypes (Zhou et al., 2019). Genes encoding the iron siderophore enterobactin (Raymond et al., 2003) were universally present, and the prevalence of the *iss* gene encoding an increased serum survival (Johnson et al., 2008) was also high.

Based on its virulence gene array containing S fimbria (Dobrindt et al., 2001) and on being a member of ST405, strain ML2/9C was categorized as ExPEC, with strains of this ST being reported as emerging uroseptic pathogens (Chowdhury et al., 2019). Strains of this pathotype are rarely reported from cattle (Bélanger et al., 2015; Schmidt et al., 2015).

The high number of prophages harbored by the sequenced genomes support the notion that these MGEs play a significant role in the evolution and genomic variability of intestinal *E. coli* pathotypes (Ogura et al., 2009; Shaaban et al., 2016). It must be noted however, that as we worked with draft genomes, probably not all prophage regions were identified in the genomes. This might be due to the fact that some phages are highly homologous in certain regions and assembling programs cannot distinguish between these regions but assemble these different regions into one single contig.

The distribution of antimicrobial resistance genes among the strains underlines the notion that these determinants are in circulation among bovine zoonotic *E. coli*, (Kennedy et al., 2017; Um et al., 2018) which can probably be attributed to the pressure of antimicrobials applied against animal pathogens (Hammerum and Heuer, 2009). The number of strains harboring antibiotic resistance genes was comparably low (5/26 of sequenced strains), probably owing to the banning of their use as growth-promoters in 2006 by the European Union. Only the ExPEC strain of bovine origin, ML2/9C showed a multidrug-resistance genotype, harboring genes encoding resistance toward eight different classes of antibiotics, including the ESBL gene *bla*_CTX-M-15_.

MLST analysis showed that all O157:H7 strains, regardless of pathotype, belonged to ST11, which is in line with earlier findings (González-Escalona and Kase, 2017; Galarce et al., 2021), while the EHEC O26:H11 strains represented ST21, which is also typical for strains of these sero- and pathotype (Abdalhamid et al., 2019; Galarce et al., 2021). A remarkable finding was that one bovine STEC, as well as two bovine and one human aEPEC strain belonged to ST10, which contains strains of various sero- and pathotypes, and is frequently isolated from human and animal sources (Manges et al., 2015).

We also attempted to place our strains on a global phylogenetic relationship of STEC and aEPEC strains, using a core-genome-based approach and the sequence set published by Arimizu et al. (2019). We found that the placement of bovine strains on the tree was generally aligned with their isolation source, and strains isolated from the same farm (i.e., the “KP” or “ML6” strains) tended to cluster together (Figure 1). The strains of human origin, however, were placed on clades with predominantly bovine isolates. This notion can be indicative of their close geographic origin, the genomic variability of the pathotype in general, as well as the zoonotic risk posed by the bovine strains isolated in our study.

In conclusion, we determined the WGS of 22 *E. coli* strains representing the STEC, EHEC, aEPEC and ExPEC pathotypes, both of human and bovine origin from Hungary, together with four commensal *E. coli* from bovine sources. Our results underline the pathogenic potential of bovine strains to humans, as EHEC and aEPEC strains of human origin as well as several bovine strains of the same pathotype harbored very similar virulence gene sets. The phylogenetic position of several human strains within the “bovine-like” lineages corroborates this notion. Several strains harbored Stx prophages with unusual combinations of integration sites. Comparative investigation of *stx-*negative, LEE-negative strains revealed the carriage of a small number of virulence genes, as well as a potential to carry virulence-related MGEs. The prophage content of the strains showed various profiles, with several widespread prophages with functions yet to be identified. Investigating the inducibility of prophages could be important toward understanding their role in the dissemination of virulence and fitness genes, as well their role in determining architecture of various STEC and aEPEC lineages within cattle populations.

## Data Availability Statement

The datasets presented in this study can be found in online repositories. The names of the repository/repositories and accession number(s) can be found at: https://www.ncbi.nlm.nih.gov/, PRJNA764596, SAMN21509413-SAMN21509438, and SRR15970245-SRR15970246.

## Author Contributions

IT and TC conceived and outlined the study. DS performed strain isolation and preliminary genetic characterization and participated in the genomic analysis as well. TM provided strains and performed serotyping. LF performed sequencing and bioinformatical analysis. DS, LF, and IT wrote the manuscript. All authors contributed to the article and approved the submitted version.

## Funding

This work was supported by the National Research, Development and Innovation Office (NKFIH), grant number K 124335 and the Bundesministerium fuer Bildung und Forschung (BMBF, Germany) within the German Center for Infection Research (DZIF; grant nos. 8032808818 and 8032808820 to TC) and the State Ministry of Higher Education, Research and Arts of the state Hessen (HMWK) through the HuKKH project (Hessisches universitaeres Kompetenzzentrum Krankenhaushygiene). DS was supported by the János Bolyai Research Scholarship of the Hungarian Academy of Sciences.

## Conflict of Interest

The authors declare that the research was conducted in the absence of any commercial or financial relationships that could be construed as a potential conflict of interest.

## Publisher’s Note

All claims expressed in this article are solely those of the authors and do not necessarily represent those of their affiliated organizations, or those of the publisher, the editors and the reviewers. Any product that may be evaluated in this article, or claim that may be made by its manufacturer, is not guaranteed or endorsed by the publisher.

## Footnotes

^1^ https://ezclermont.hutton.ac.uk/

## Supplementary Material

Supplementary Table 1 Genome sequences used for the phylogenetic analysis of the strains with WGS determined in the current study.

Supplementary Table 2 List of virulence genes identified by VirulenceFinder and VFDB in the WGS of strains determined in the current study.

Supplementary Table 3 Prophages or prophage-like sequences identified by PHASTER in the WGS of strains determined in the current study.
